# Assessment of the Usefulness of Multiplex Real-Time PCR Tests in the Diagnostic and Therapeutic Process of Pneumonia in Hospitalized Children: A Single-Center Experience

**DOI:** 10.1155/2017/8037963

**Published:** 2017-01-15

**Authors:** Ewelina Gowin, Alicja Bartkowska-Śniatkowska, Katarzyna Jończyk-Potoczna, Joanna Wysocka-Leszczyńska, Waldemar Bobkowski, Piotr Fichna, Paulina Sobkowiak, Katarzyna Mazur-Melewska, Anna Bręborowicz, Jacek Wysocki, Danuta Januszkiewicz-Lewandowska

**Affiliations:** ^1^Department of Family Medicine, University of Medical Sciences, Poznan, Poland; ^2^Department of Paediatric Anaesthesiology and Intensive Therapy, University of Medical Sciences, Poznan, Poland; ^3^Department of Paediatric Radiology, University of Medical Sciences, Poznan, Poland; ^4^Department of Medical Diagnostics, University of Medical Sciences, Dobra 38, Poznan, Poland; ^5^Department of Paediatric Cardiology, University of Medical Sciences, Poznan, Poland; ^6^Department of Paediatric Diabetology, University of Medical Sciences, Poznan, Poland; ^7^Department of Paediatric Oncology, Hematology and Bone Marrow Transplantation, University of Medical Sciences, Poznan, Poland; ^8^Department of Infectious Diseases, University of Medical Sciences, Poznan, Poland; ^9^Department of Paediatric Pulmonology, University of Medical Sciences, Poznan, Poland; ^10^Department of Health Promotion, University of Medical Sciences, Poznan, Poland; ^11^Department of Molecular Pathology, Institute of Human Genetics, Polish Academy of Sciences, Poznan, Poland

## Abstract

The aim of the study was assessment of the usefulness of multiplex real-time PCR tests in the diagnostic and therapeutic process in children hospitalized due to pneumonia and burdened with comorbidities.* Methods*. The study group included 97 children hospitalized due to pneumonia at the Karol Jonscher Teaching Hospital in Poznań, in whom multiplex real-time PCR tests (FTD respiratory pathogens 33; fast-track diagnostics) were used.* Results*. Positive test results of the test were achieved in 74 patients (76.3%). The average age in the group was 56 months. Viruses were detected in 61 samples (82% of all positive results); bacterial factors were found in 29 samples (39% of all positive results). The presence of comorbidities was established in 90 children (92.78%). On the basis of the obtained results, 5 groups of patients were established: viral etiology of infection, 34 patients; bacterial etiology, 7 patients; mixed etiology, 23 patients; pneumocystis, 9 patients; and no etiology diagnosed, 24 patients.* Conclusions*. Our analysis demonstrated that the participation of viruses in causing severe lung infections is significant in children with comorbidities. Multiplex real-time PCR tests proved to be more useful in establishing the etiology of pneumonia in hospitalized children than the traditional microbiological examinations.

## 1. Introduction

Acute respiratory tract infections are the most common infectious diseases among children. The incidence of community-acquired pneumonia in Europe is 33/100,000 in the age group < 5 years [1]. Its clinical manifestation includes symptoms ranging from mild rhinitis to severe pneumonia leading to respiratory failure. The risk group of severe disease course includes children < 5 years of age, especially boys, individuals in immunosuppression, and patients suffering from chronic diseases [2]. Establishing the etiological factor is a difficult task. There are no clinical, radiological, or biochemical markers that would enable the differentiation between bacterial and viral infections [3]. This frequently results in the overuse of antibiotics in the therapy of acute respiratory infections. British, American, and Polish guidelines state that, in children hospitalized due to pneumonia, microbiological examinations should include blood cultures, the detection of the presence of viruses with the use of PCR (Polymerase Chain Reaction) or immunofluorescence in material collected from the nasopharynx (smear or upper respiratory aspirate), the assessment of antibodies against* Mycoplasma* and* Chlamydophila* in classes IgM and IgG, and the comparison of antibody levels in the acute phase of the disease and during convalescence [4–6].

In children it is difficult to collect reliable material for microbiological analysis in a low or noninvasive manner and retain its representativeness of the lower respiratory tract flora. Nasal smear cultures are not useful in establishing the etiology of pneumonia. The pathogens grown in a such manner include both physiological flora as well as flora that could potentially cause pneumonia. On the other hand, microbiological cultures need to be secured before antibiotic therapy is commenced, which is impossible in many cases. The solution to this problem is the detection of microbial genetic material in, for example, the material acquired from nasal smear cultures.

It is, however, important to remember that PCR results may be positive in the case of persistent infections; in some pathogens prolonged shedding is observed even up to 7 months since the beginning of an infection [7]. Another problem is that traditional bacterial cultures are insufficient to establish the etiology, mainly due to the significant participation of viruses in the etiopathogenesis of these infections. This especially concerns children in their first year of life, in whom viral infections may be responsible for even 67% of pneumonia cases [8]. The currently used molecular examinations enable quick identification of numerous pathogens. Detecting the influenza virus with PCR is a widely accepted and utilized method of confirming infection [9]. This is also true for the respiratory syncytial virus (RSV). The main drawback of tests detecting just a single pathogen is the necessity of requesting every examination separately, collecting samples for analysis multiple times, and making decisions concerning the selection of pathogens for analysis. Test panels used for establishing the presence of the most important bacterial and viral pathogens enable simultaneous detection of the most significant etiological factors. A separate problem is that, apart from the accepted viral etiology, pneumonia may also be caused by new viruses, such as the human metapneumovirus (hMPV), human coronavirus, or bocavirus [10]. Molecular techniques are more sensitive and capable of diagnosing more viruses [11]. Prompt and accurate diagnosis is important for infection control and surveillance, patient cohorting, treatment choices, and avoiding antibiotics. Previous experience with the use of the multiplex real-time PCR tests in the population of children is limited [11–14].

 Aim of the study is the assessment of the usefulness of multiplex real-time PCR tests in the diagnostic and therapeutic process in children hospitalized due to pneumonia and burdened with comorbidities.

## 2. Material and Methods

The study group was composed of children hospitalized due to pneumonia in the period between 01.2014 and 02.2015 at the Karol Jonscher Teaching Hospital in Poznan, in whom multiplex real-time PCR tests (FTD respiratory pathogens 33; fast-track diagnostics) were used in the diagnostic process. FTD respiratory pathogens 33 is an in vitro test with eight multiplex real-time PCR reactions for the qualitative detection of the following viruses, bacteria, and fungi causing respiratory infections: influenza A, B, and C; parainfluenza viruses 1, 2, 3, and 4; coronaviruses NL63, 229E, OC43, and HKU1; human metapneumoviruses A and B; rhinovirus; respiratory syncytial viruses A and B; adenovirus; enterovirus; parechovirus; bocavirus; cytomegalovirus;* Pneumocystis jiroveci*;* Mycoplasma pneumoniae*;* Chlamydia pneumoniae*;* Streptococcus pneumoniae*;* Haemophilus influenzae* type B;* Staphylococcus aureus*;* Moraxella catarrhalis*;* Bordetella* spp. (except* Bordetella parapertussis*);* Klebsiella pneumoniae*;* Legionella* species; and* Salmonella* species.

Indications for the examination included

- radiologically confirmed severe pneumonia nonresponding to standard therapy,
- the presence of complications, for example, respiratory failure,
- the presence of comorbidities,
- impaired immunity.

The analysis included the following factors: age, sex, comorbidities, immunosuppression, low body mass, airways obstruction, and respiratory failure requiring admission to ICU (Intensive Care Unit).

Respiratory samples (throat or nasal swabs) were collected from all patients. The next step was extraction of pathogens' genetic material either DNA or RNA, followed by amplification of specific regions by real-time polymerase chain reaction. The presence of specific pathogen sequence in the reaction is reported as a cycle threshold value.

Apart from the multiplex real-time PCR method, also traditional microbiological culture tests were performed in the studied children: blood cultures, nasopharynx smear cultures, and respiratory aspirate cultures. Positive microbiological cultures were defined a ≥105 CFU/mL.

All the described tests were done as diagnostic tests during hospitalization. Informed consent was obtained from parents/legal guardians on admission to hospital.

### 2.1. Statistical Analysis

Data were presented as percentages or medians and range means and standard deviations. Interval data were analyzed by Mann–Whitney test since data did not follow normal distribution (Kolmogorov-Smirnov test). Nominal data were analyzed by chi-square test of independence or in case of zero observed frequencies an exact Fisher-Freeman-Halton test was used. Data were analyzed with the use of statistical packages Statistica 10 (StatSoft, Inc.) and StatXact 8.0 (Cytel); all tests were considered significant at *p* < 0.05.

## 3. Results

The study group included 97 patients, 52 boys and 45 girls; the average age in the group was 56 months. The presence of comorbidities was established in 90 children (92.78%), including respiratory system diseases in 25 cases, cardiac diseases in 21 cases, neurologic diseases in 13 cases, and neoplastic diseases in 9 cases. Immunosuppression was identified in 11 patients (11.34%). The details are provided in Table 1.

Positive test results were achieved in 74 patients (76.3%). The presence of a viral factor was established in 61 samples (82% of all positive results); bacterial factors were found in 29 samples (39% of all positive results). The presence of the genetic material of a single pathogen was found in 34 children (45% of all positive results). The details of the analysis are presented in Table 2.

On the basis of the obtained results, 5 groups of patients were established:

Viral pneumonia: 34 patients
Bacterial pneumonia: 7 patients
Pneumonia of mixed etiology: 23 patients
Pneumocystis pneumonia: 9 patients
Pneumonia of unestablished etiology: 24 patients

When comparing these groups, no differences were found in terms of age, day of material collection in relation to hospitalization time, or the necessity of ICU stay. Radiological image analysis was not sufficient to unambiguously diagnose the etiology of pneumonia or respiratory infection. The presence of airflow obstruction was significantly higher in patients with a viral etiology (*p* = 0.0011).

On admission 54% of children with negative real-time PCR results had elevated procalcitonin levels. This proportion is significantly higher when compared to patient with established etiology (*p* = 0.0186). Such difference was not observed while comparing proportion of patients with markedly elevated CRP levels (>50 mg/dL). The prevalence of increased procalcitonin levels (>2 ng/ml) was higher in patients without established etiology. The difference is of statistical significance. Such differences were not observed when comparing number of patients with increased CRP levels.

Airflow obstruction was found in 44 patients (45%); 75% of them had viral etiology of pneumonia. The difference was of statistical significance (*p* = 0.0011).

**Figure 1:**
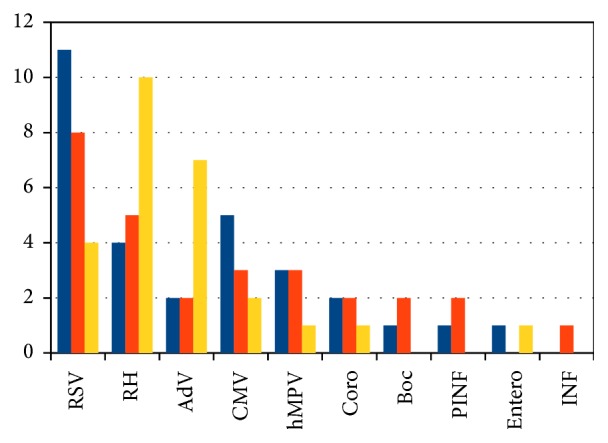
Results of nasopharyngeal samples analyzed by multiplex real-time PCR in relation to age groups. AdV: adenovirus; Boc: bocavirus; CMV: cytomegalovirus; Coro: coronavirus; Entero: enterovirus, hMPV: human metapneumovirus; INF: influenza virus; PINF: human parainfluenza virus; RH: rhinovirus; RSV: respiratory syncytial virus.

The most frequently identified pathogen was RSV in 23 children (23.7%), and rhinovirus (RH) was found in 19 patients (19.6%), adenovirus (AdV) in 11 (11.3%), cytomegalovirus (CMV) in 10 (10.3%), and* Pneumocystis jiroveci* (PNP) in 9 (9.3%). Purely bacterial etiology was established in 8 patients (8.2%). The details are presented in Figure 1. Relations were found between age and the presence of individual microorganisms: infections of RSV etiology were more frequent in younger children (median age 10.5 months versus 42 months; *p* = 0.0133); airflow obstruction symptoms were also more frequent in this group (*p* = 0.0008). In children under the age of one, the dominant pathogen was RSV, which was found in 32% of patients in this age group. 56% of all positive results for RSV occurred in children in their first year of life.

Blood cultures were performed in 75 children, in 62 cases they were sterile (82,67%), bacterial flora growth occurred in 13 cases, and in 3 (4%) cases it was pathological (*S. aureus*,* Serratia* sp., and* E. coli*). Respiratory aspirate cultures were performed in 64 children, they were sterile in 15, physiological respiratory tract flora growth was achieved in 28 cases, and in 23 it was pathological; in 8 of these cases the results were confirmed with multiplex real-time PCR tests.

Nasal smear cultures were performed in 58 children, they were sterile in 15, and the presence of pathological flora was found in 17 cases (29.31%); in 5 of these cases the results were confirmed with multiplex real-time PCR tests.

Detailed results of microbiological analyses of patients with at least one positive microbiology results are presented in Table 3. In the group of children with negative results of multiplex PCR tests, blood cultures were positive in 3 cases (*E. coli*,* Serratia*, and* S. aureus*), tracheal aspirate cultures were positive in 7 (in 4 of them pathological flora was grown), and nasal smear cultures indicated the presence of pathogenic flora in 5 cases (see details in Table 3).

Test results enabled the implementation of targeted therapy in 13 patients; details are presented in Table 4.

## 4. Discussion

The acquired results confirm the usefulness of multiplex real-time PCR tests in establishing the etiology of severe pneumonia in hospitalized children. Probable etiology of pneumonia was diagnosed in 76% of children with the use of this method. After the exclusion of children with neoplastic diseases from the group, this percentage increased to 79.54%. Similar percentages of positive results are often encountered in the literature [8, 12, 14]. Bierbaum et al. achieved positive results of multiplex real-time PCR tests detecting only viral factors in 76% of cases in a group of children below the age of six with symptoms of respiratory tract infection and the dominant pathogen was RSV [12]. In a study conducted by Mengelle et al. on a group of 914 children with symptoms of respiratory infection, 90% of the collected samples were positive, with RH, RSV, and IF (influenza virus) as the most frequent pathogens [15]. In our analysis, we found a more significant participation of RSV; however, only patients with pneumonia underwent the analysis. The general lower participation of the viral factor in comparison to the research by Mengelle most likely results from the fact that our study included a number of bacterial factors that the above-mentioned work did not take into consideration. In a Swiss study conducted by Cevey-Macherel et al. in 2009, the etiology of 85% of pneumonia cases was diagnosed in children below the age of five with the use of the PCR method; in 67% the results pointed to the presence of viruses and in 52% bacteria, and in 33% of the children the etiology was mixed [16]. In an Italian study within a group aged 5–14 years, etiology was established in 77% of cases with the use of molecular analysis. The presence of viruses was revealed in 65% of samples and bacteria in 40% of samples, while mixed etiology was present in 28% of children [17]. Mengelle et al. demonstrated that infections caused by more than one virus are common and occurred in 30% of the children included in his study.

The sensitivity of virus detection of the multiplex real-time PCR method is high and reaches almost 90% [11]. When genetic material of several pathogens is revealed with the use of the PCR method, it may result from a simultaneous infection caused by two pathogens or from an infection in a patient who is already a carrier of another pathogen. It may also result from establishing the presence of viral genetic material after a previous infection, through the so-called virus shedding. It is estimated that the etiology of pneumonia is mixed in about 30% of children [18]. Some works point out that there is a relation between the type of viral factor and the risk of coinfection. Martic et al. found coinfections with different pathogens in 50% of infections caused by adenovirus [19]. The high percentage of coinfections in our study may be explained by the specificity of the study group. The majority of the children were burdened with the presence of chronic diseases, and some of these patients were in immunosuppression. The presence of bacterial coinfection is frequent in viral etiology of pneumonia; however, bacteria are not always responsible for active infections. In children with pneumonia, establishing the presence of viruses in the upper respiratory tract is considered as a probable etiological factor. Positive results may also be achieved in individuals in temporary asymptomatic carrier state during convalescence after a previous infection [13]. It is, therefore, necessary to remain very cautious when interpreting examination results. The clinical significance of human RV RNA detection in respiratory samples remains unclear [20]. The PCR method does not enable unambiguous differentiation between infection and carriage, even with the use of the quantitative method. It needs to be stated, however, that comparative studies of carriage significantly more often indicated the presence of* S. pneumoniae* in children with pneumonia compared to the healthy population [21]. The available literature contains reports stating that higher numbers of viruses were usually associated with milder disease course [20]. The exception was the coinfections with RSV and hMPV [22]. Infections of this type are characterized by intensified obstruction symptoms [22]. In our group, the children with infections caused by mixed pathogens did not differ from the children with infections caused by single pathogens, in terms of both the severity of disease course and intensity of inflammatory reaction.

The high percentage of positive results of examinations detecting viral genetic material points to the significance of viruses in the occurrence of pneumonia in children. The literature indicates that viruses may be responsible for the majority of pneumonia cases in children [8, 16]. In our group, in the case of hospitalized children, some patients received outpatient antibiotic therapy, which was then continued during inpatient care in all cases. Therefore, all samples were collected during antibiotic therapy, on the fifth day on average. Hence, it is not possible to completely exclude the presence of bacterial factors, which were not identified in the upper respiratory tract later on due to the ongoing antibiotic therapy. This may explain the relatively low percentage of bacterial genetic material detected in the group we analyzed. The current guidelines recommend the use of antibiotic therapy in children hospitalized due to pneumonia, but the lack of clinical improvement despite the application of broad-spectrum antibiotic therapy may suggest a viral cause of infection [4–6].

The studied group was unique due to the high participation of patients with comorbidities, some of them in immunosuppression. Such patients are at a higher risk of severe course of infection and prolonged hospitalization. The diagnosis of a viral etiology of infection enables moderate use of antibiotics and allows for more emphasis on symptomatic treatment, which is the most beneficial for such patients. Because viruses are easily transmitted from patient to patient, advanced infection control methods are critical in controlling the spread of viruses [20]. RSV turned out to be the most frequently detected pathogen, especially in the group of children under the age of one. Moreover, it was frequently the only identified pathogen. In children from risk groups, RSV causes infections with severe course, and it is characterized by high infectiousness. Diagnosing RSV as the etiological factor enables the isolation of or cohorting the patients in order to reduce the spread of the infection and the implementation of the recommended management for severe obstruction [4, 5]. Research is currently being conducted on the implementation of antiviral drugs which are more effective than the previously used ribavirine [23–25]. In the case of viral infections such as influenza, the establishment of the etiological factor enables the implementation of specific treatment. In the studied group, a single case of influenza was found, but the year in which the study was conducted was not an epidemic year. Molecular tests for detecting the influenza virus only are widely applied. They are very useful during epidemic seasons, but it is important to remember that the disease may occur also outside such periods. In certain situations, it may be difficult to select a diagnostic test only on the basis of symptoms. Moreover, in the case of influenza, the time of treatment implementation is crucial. Therefore, including influenza in the standard diagnostic set prevents the omission of this important infection factor.

The conducted analysis showed limited usefulness of traditional microbiological examinations in the diagnostic process of pneumonia. Taking blood cultures is also considered standard in severe pneumonia, but its usefulness is not significant. In a group of patients with radiologic confirmed pneumonia, Esposito revealed the presence of* S. pneumoniae* in 14.3% of cases; 91.8% of the diagnoses were based on positive results of real-time PCR tests conducted on material collected from the respiratory tract, while positive results of blood cultures were present only in 8.14% of cases [22]. Nasopharyngeal swabs cultures often show flora growths that may constitute colonization. In our analysis, traditional microbiological examinations revealed the presence of potentially pathogenic bacteria in material collected from 31 children; in 11 of these cases there was agreement with the results of multiplex real-time PCR tests. In 8 patients it was not possible to assess the agreement of research methods due to the fact that in the FTD respiratory pathogens 33 test microorganisms such as* Pseudomonas aeruginosa*,* Escherichia coli*, or* Serratia* sp. are not detected.

The majority of previous works assessed the results of multiplex real-time PCR tests conducted in admission rooms on all patients reporting with respiratory symptoms [15–17]. Because of the costs associated with these tests, it is difficult to use them in such a manner in daily practice. The mentioned analyses seem to be more epidemiological in nature. In our work we made an attempt to assess the usefulness of tests which were conducted during hospitalization in children with severe pneumonia not responding to standard therapy. In this group of patients, the benefits of the applied treatment were satisfactory in relation to the diagnostic cost incurred. The confirmation of viral etiology was very important, especially in the group of children at a risk of severe course of infection. In such a unique group of patients, constituted by children burdened with comorbidities, under immunosuppression, and after marrow stem cell transplants, the implementation of targeted treatment should be taken into consideration with the use of modern antiviral drugs, as well as such steps as isolation and the reduction of immunosuppression. In the guidelines of the neutropenia management it is strongly advised to isolate patients with documented respiratory viruses until symptoms resolve [20].

There are some limitations to our study. We have collected a heterogenic group of patients (different indication for testing, different comorbidities, and chronic diseases). The number of patients and the number of specimens were too small to perform elaborate statistical analysis. Sample collection for microbiological cultures was incomplete. The exact timing of sample collection relative to antibiotic administration was not accurately documented. Similar problems were described in other clinical studies in this subject topic.

Our analysis demonstrated that coinfection with viruses is common in severe lung infections in children with comorbidities. Multiplex real-time PCR tests proved to be more useful in establishing the etiology of pneumonia in hospitalized children than the traditional microbiological examinations widely used in the applicable diagnostic process to date.

## Figures and Tables

**Table 1:** Characteristics of study subjects.

Number of patients	97
Mean age (months)	56.3
Male/female ratio	52/45
ICU admission	53 (54%)
Airways obturation	44 (44%)
Coexisting diseases	90 (92.78%)
Heart diseases	21 (20%)
CHD	17
HF	2
HA	1
Neurologic diseases	13 (13%)
Cerebral palsy	7
Epilepsy	4
Myopathy	2
Neoplastic diseases	9 (9%)
ALL	6
Solid tumor	3
Pulmonary diseases	12 (12%)
Asthma	8
CF	4
Immunosuppression	11 (11%)
Malnutrition	41 (42%)
FTD 33 and microbiology results	
FTD 33+/microbiology−	63 (65%)
FTD 33+/microbiology+	11 (11%)
FTD 33−/microbiology+	6 (6%)
FTD 33−/microbiology−	17 (18%)

ALL: acute lymphoblastic leukemia, CF: cystic fibrosis, CHD: congenital heart defect, FTD 33−: FTD respiratory pathogens 33 test negative results, FTD 33+: FTD respiratory pathogens 33 test positive results, HA: hypertension, HF heart failure, ICU: Intensive Care Unit, microbiology: microbiological cultures negative results, and microbiology+: microbiological cultures positive results.

**Table 2:** Prevalence of pathogens and rates of codetection.

	Single pathogen	Bacterial coinfection	Viral coinfection	Coinfection with PNP	Mixed coinfection viral and bacterial	Total number
*H. influenzae*	0	1	11	0	2	14
*S. pneumoniae*	0	2	3	0	3	8
*S. aureus*	3	3	2	0	3	11
*Klebsiella*	0	0	1	0	1	2
*Moraxella*	0	0	2	0	1	3
*Mycoplasma *	1	0	0	0	0	1
*Legionella*	0	0	1	0	1	3
RSV	11	4	6	1	1	23
RH	5	4	2	1	7	19
AdV	3	1	2	0	5	11
hMPV	3	0	2	0	2	7
CMV	1	1	2	2	4	10
INF	0	1	0	0	0	1
ENTERO	1	1	0	0	0	2
PINF	1	0	2	0	0	3
CORO	1	2	0	0	2	5
BOC	1	1	0	1	0	3
PNP	4	0	4	0	1	9

AdV: adenovirus; BOC: bocavirus, CMV: cytomegalovirus, CORO: coronavirus, Entero: enteroviruses, hMPV: human metapneumovirus, INF: influenza virus, PINF: human parainfluenza virus; PNP: *Pneumocystis jiroveci*, RH: rhinovirus; and RSV: respiratory syncytial virus.

**Table 3:** Results of diagnostic test in patients with at least one positive microbiology culture.

Blood culture	Nasal swab	Respiratory tract aspirate	PCR
Positive blood culture			
*S. aureus*	Sa	Pseud	0
*E.coli *	Sa	X	0
*Serratia*	Pseud	Pseud	0
		Positive respiratory tract aspirate	
0	X	Sa	RH/AdV/Sa
X	X	Sp	RH/Sp
0	X	Hinf, Klebs	RH/Hinf
0	X	Hinf	RSV/Hinf
0	X	Hinf	RH/AdV/Hinf
0	F	*E.coli*	RSV/CMV/Hinf
X	X	Hinf	PINF/Sa
0	0	Sa	RSV
X	F	Klebs	RSV
0	F	Sp	RSV
0	F	Pseud	hMPV
X	X	Klebs	AdV/PINF
		Hinf	0
0	F	Sa	Sa
	Positive nasal swab		
0	Sa	0	0
X	Sa	F	RSV
X	Sa	X	RH
X	Pseud	X	Sa
0	Sa	0	Sa
X	Sa	F	CORO/Sa
0	Sa	F	Sa/INF
	Positive nasal swab and respiratory tract aspirate		
0	Sa	ESBL	0
0	Sa	Sa	RH/PNP
0	Sa	Hinf	RSV/hMPV
0	Sp	Pseud, Sp	CMV
0	Pseud	Serr, Sa	hMPV
X	Sa	Sa	Sa/Sp
0	Sp	Sp	BOC/Sp

AdV: adenovirus; BOC: bocavirus; CMV: cytomegalovirus; CORO: coronavirus; *E. coli*: *Escherichia coli*; F: physiologic flora; Hinf: *Haemophilus influenzae*; hMPV: human metapneumovirus; Klebs: *Klebsiella pneumoniae*; PINF: human parainfluenza virus; PNP: *Pneumocystis jiroveci*; Pseud: *Pseudomonas aeruginosa*; RH: rhinovirus; RSV: respiratory syncytial virus; Sa: *Staphylococcus aureus*; Sp: *Streptococcus pneumoniae*; X: test not done; and 0: negative results.

**Table 4:** Targeted therapy implemented based on FTD33 positive results.

FTD33 results	Influenza	*Mycoplasma pneumoniae*	* Legionella*	*Pneumocystis jiroveci*
Number of patients	1	1	2	9
Targeted treatment	Oseltamivir	Macrolides		Trimethoprim/ sulfamethoxazole
